# Analyzing housing supply location choice: a comparative study of the modelling frameworks

**DOI:** 10.1038/s41598-024-51754-9

**Published:** 2024-01-16

**Authors:** Yu Zhang, Eric J. Miller

**Affiliations:** Department of Civil and Mineral Engineering, University of Toronto, 35 St George St, GB305, Toronto, ON M5S 1A4 Canada

**Keywords:** Civil engineering, Environmental social sciences, Mathematics and computing

## Abstract

The purpose of this study is to predict the location of new housing supply and compare two different modelling frameworks. Housing supply significantly influences land use simulations in urban microsimulation systems, closely linked with demographic, transportation, and environmental modules. The supply of new dwellings in urban simulation models have evolved from static, exogenous inputs to dynamic, agent-based determinations. This study follows this trend to examine two approaches to modelling the spatial distribution of new housing supply: the first approach models the development choice of each location; the second approach models the location choice of each residential project. Multinomial logit and nested logit models are applied to a Toronto empirical dataset. The results show that although the first approach achieves higher goodness-of-fit and prediction accuracy, the second approach performs better in explaining the locational preference of individual projects. Project characteristics such as structure type and construction cost, as well as location characteristics such as housing price, number of sales, and population density affect the spatial distribution of new housing supply. Both approaches are evaluated regarding estimation, prediction, and microsimulation system integration. The findings enhance housing modelling literature and inform urban microsimulation’s housing supply model configuration.

## Introduction

As elsewhere globally, North American urban regions have experienced massive housing property development over the past few decades. In response to advancements in transportation modes and changes in urban industrial structure, residential neighbourhoods have gradually expanded from the downtown to lower density suburban regions. The current spatial distribution of residential housing in the Greater Toronto Area shows a pattern in which the downtown contains both low-rise old houses and high-rise new condo apartments, and the suburbs have mostly been developed into detached houses and townhouses, with condo apartments built along the major transit lines. As the urban form has evolved, so has the site selection process and determinants of housing project location choices. Many housing market models have not sufficiently considered the site selection of housing developers even though households’ location choices have been extensively studied. To build a complete and holistic modelling system of the residential housing market, it is important to also understand the site selection process of builders.

This paper explores two approaches to modelling the location choice of residential developers that are common in the literature (but rarely directly compared): the first approach represents development decisions being made by each location choosing the type of development to occur at that location (including no development); the second approach models the location choice of individual developers from a set of possible locations. Despite that the two approaches both generate the same outcome (prediction of new residential development by type and location), they assume different representations of the choice process, and each have pros and cons to consider when it comes to treating spatial relationships among residential development, construction capacity of the location, and the control of total supply. This paper presents, discusses, and compares the two approaches to modelling housing supply location choices, based on an empirical study in the City of Toronto. It provides insight into the implementation of housing supply location models in future overall models of housing markets.

The rest of the paper is organized as follows. “Literature review” section reviews the current research on location choice modelling applying the two approaches. “Methodology” section refines the research question and proposes the study framework and methodologies. “Data sources and data preparation” section describes the study area, the City of Toronto, and the data employed in this study. “Empirical results of approach 1: modelling the development choice of locations” section presents the results of the first approach, modelling the development choice of each location, and analyzes in detail the model framework, goodness of fit, coefficient estimation, elasticity of variables and the model prediction and validation. The results of the second approach, discrete choice modelling of developers over large sets of location alternatives, are presented in “Empirical results of approach 2: modelling the location choice of residential projects” section. “Comparison of the two approaches” section compares the two modelling approaches in terms of data requirements, parameter estimation, prediction performance and potential for usage in urban microsimulation systems. The paper concludes with a discussion and summary of the study’s findings.

## Literature review

### Residential development choice modelling of locations

The modelling of residential development in some integrated urban simulation models takes the first approach described above and models the housing supply through land use change simulation. Residential development is modelled as the results of the development potential or choice of the location itself. A cellular automata model was applied in simulating the residential development in Guangzhou by Li and Liu^1^. Logit models were frequently applied in the land use change simulations. Haider^2^ models the house type choice of the zones in the Greater Toronto and Hamilton Area. The real estate development model in early versions of UrbanSim is structured to predict the probability for the grid cell to experience a development event within the simulation year^3^. Variables in site characteristics, urban design-scale, regional accessibility and market conditions are used in the residential development model, with user-specified constraints on the type of development allowed in the grid cell. PECAS^4^ models the aggregate allocation of space by building category of each land use zone with a set of logit allocation models. SILO^5^ models the developer’s behaviour associated with the dwelling object and simulates the events of new construction, demolition, renovation and deterioration by location.

### The location choice modelling of residential developers

The second approach described above has few applications in the modelling of residential development. The discrete location choice modelling of agents is more commonly applied on the demand side, such as housing location^6^, firm location^7,8^, job location^9^, school location^10,11^, etc. The choice set is formed based on random selection, importance sampling, or universal set. In terms of the housing supply location choice of developers, the choice set is largely constrained by local zoning, and developers have limited options when locating their new housing projects. The location of new residential areas in Netherland is modelled by Rietveld and Wagtendonk^12^ using logit models. The area is divided into cells and the probability or suitability of residential development is formed based on proximity, accessibility and density factors. Residential developers make site selection choice based on site suitability. Haider and Miller^13^ developed models for the location choice of developers of different dwelling types at zonal level. Developers choose the alternative location from a reduced choice set. Li et al.^14^ developed a GIS-based site selection system for real estate projects. All available sites are evaluated through data envelopment analysis based on multiple criteria.

Residential location choice has been more broadly modelled from the demand side than the supply side. However, household preference on the locational attributes of the dwelling units also influences the site selection of residential developers. Accessibility, attributes of the dwelling units, and the demographic characteristics of the household are generally included in modelling the housing location choice^15–22^. The demand side research commonly applies the second approach; i.e., households’ choices are modelled from a large set of dwelling units or locations as the alternatives. The utility-based logit model is usually applied, and the choice set is formed based on certain sampling methods.

Though empirical studies using the second approach commonly have issues in low prediction accuracy, the approach reveals the preference of the decision maker and the influence of each explanatory variable on the probability of each alternative to be chosen. This study follows current research applying the two modelling approaches, and compares them in modelling the housing supply to provide modellers with comprehensive insights in analyzing the residential supply market.

## Methodology

### Define the question and select the influential factors

The research question of this paper is how to model the locational distribution of new residential housing supply. On the one hand, the site selection process by developers/builders is a complicated one. The project proposal considers the attributes of the selected land parcel and its surrounding area to estimate the profit potential. A company’s budget, experience and knowledge are also considered. A project’s feasibility varies based on zoning regulations, land availability, and other regulations. The site choice of multiple developers constrained by zoning leads to the current spatial distribution of housing supply. On the other hand, the development potential of the land parcel itself heavily influences its future land use that is likely to arise. The land parcels near transit lines with dense economic activities, for example, may have a probability of being developed into high-rise condo apartments due to their geography and land features, regardless of which developer acquires the land and develops it. Thus, the research question can be refined as: how to model where a proposed residential housing project locates over space? Or, equivalently, how to estimate the development potential of a certain location?

**Figure 1:**
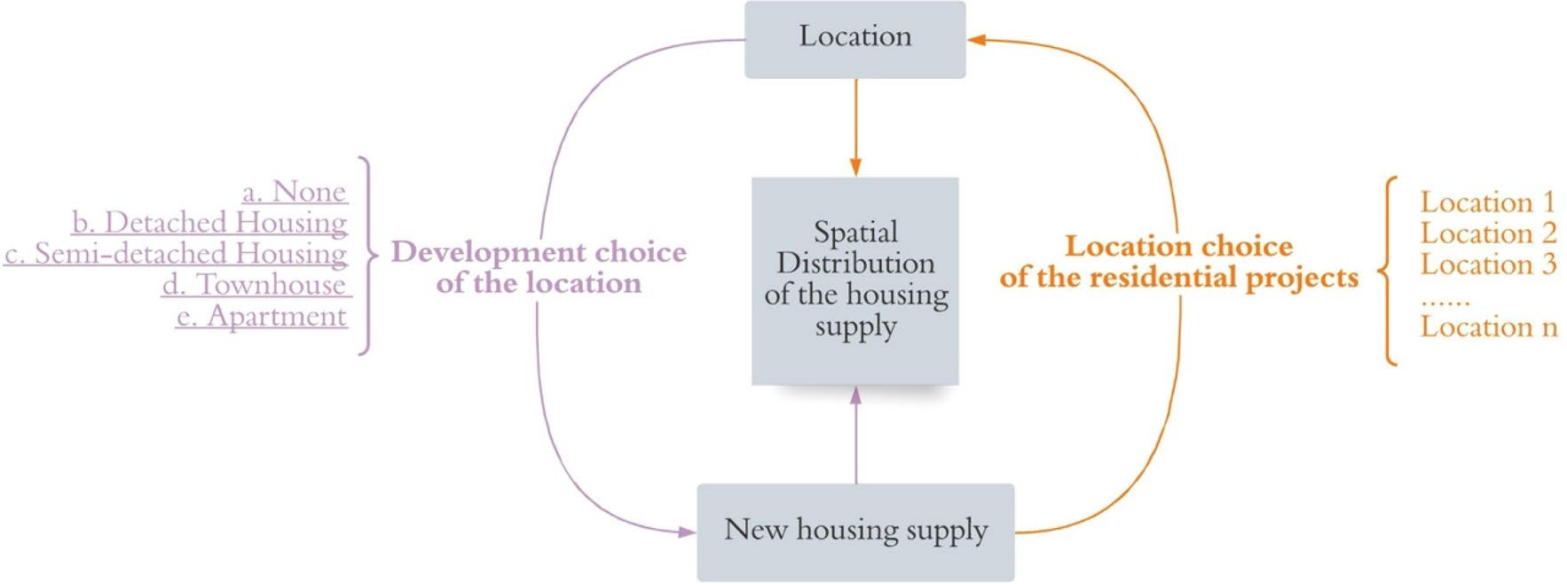
Analysis Framework: two approaches.

As illustrated in Fig. 1, the above two research questions frame the spatial distribution of residential supply in two different ways, one with the decision-making unit as the developer, the other as the location itself, which leads to two different modelling approaches. The first approach models the “development choice” of each land parcel, whereby the choices of each land parcel to be developed into single detached housing, semi-detached housing, townhouses or condos, or not to be developed, account for the residential supply distribution in the city. This approaches can be defined as the “location-conditional development type choice” approach. However, this approach ignores the spatial path dependent influence between each land parcel, and overall development capacity constraints. I.e., in this modelling approach, it may happen that no location decides to have development, or, alternatively every location decides to do something. The problem can be mitigated by a two-tiered decision-making process, with the first layer being a binary decision on whether or not to build residential units, and the second layer being the type of dwelling to choose.

The second approach models the location of a proposed project. Accordingly, the location choice could be made based on the project’s attributes, such as the number of homes to be created and the estimated construction costs. This approaches can be defined as the “development location choice” approach. While arguably a more “behavioural” approach to the problem, since it is developers that choose what to build where and when, at least three issues exist with this approach. Firstly, it allows multiple projects to have the same location choice, and the projects are not “talking” to each other, which could lead to the chosen location exceeding its development capacity. Second, due to the large choice set, the most desirable alternative would be predicted with a low probability of being chosen, and it is difficult to correctly identify the actual chosen location, resulting in a lower prediction accuracy. Thirdly, the large choice set creates computational burdens for the model as the attributes of every alternative will be included. In addition, the second approach cannot perform well when the database concerning the proposed projects is challenging to obtain or simulate.

The two models are developed under a strong assumption that the developable land (The developable land means excluding land parcels that are fully occupied by waterbodies, designated greenspace, agricultural land, or industrial land. Further data cleaning (removing the locations with zero average dwelling value and zero average rent, and with no transaction records during the modelling time period) is performed before the samples are used in the model.) is always available and that builder’s attributes are the same. Although unrealistic, it is hoped that the analysis can provide a cornerstone for developing realistic dynamic models that allows future extension to accommodate the influence of zoning and builder’s attributes.

The variables to be included in the utility function can be drawn from the literature in three aspects: locational factors, density, and market factors. Centrality and accessibility affect the development potential and will be considered by the developers for the site location choice. The density of economic activities, housing transactions, and population and jobs will also influence the attractiveness of the location. The number of transactions, and average selling price of the housing units of different dwelling types, will influence the choice of dwelling type of the project. To reduce the effect of intercorrelation among the explanatory variables, correlation tests and variable selection were conducted. The variables included in the models are, the average sales price of new housing supply, and number of transactions, distance to the CBD, road density, average household income, percentage of auto as the commuting mode, percentage of park, open area, residential area, job accessibility by car.

### Meta-algorithms of the two approaches

**Figure 2:**
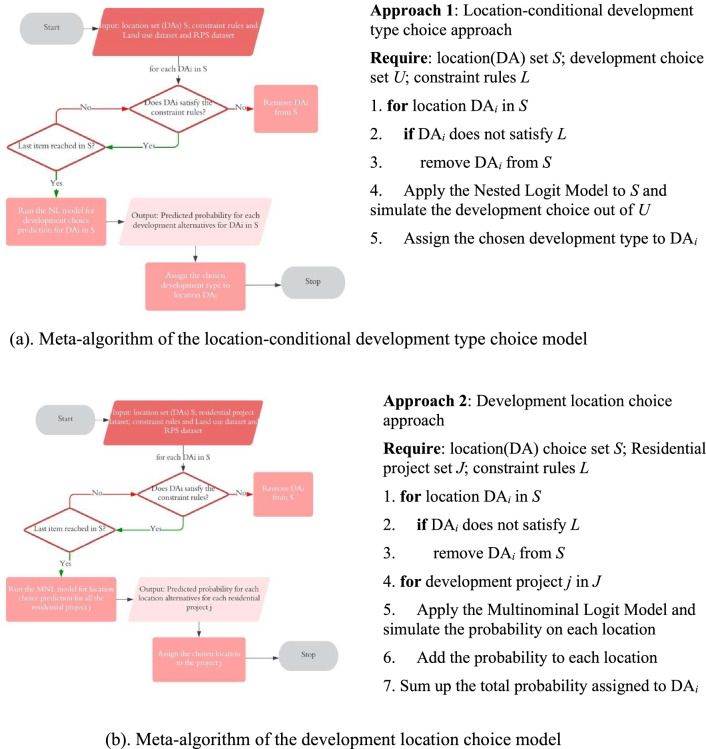
(**a**) Meta-algorithm of the location-conditional development type choice model. (**b**) Meta-algorithm of the development location choice model.

The meta-algorithms of the two approaches are further elaborated below and in the flowchart in Fig. 2. The first step of both approaches is filtering out the infeasible locations. Ideally the overlap between feasible set and aware set should be used. However, it would be impossible to know the aware set of each residential project. Thus this study uses several constraints to narrow down the universal set and tries to form the feasible set. The spatial unit is Dissemination Area (DA) in this study and all the DAs are asked whether they satisfy the land use and housing transaction constraints. The specific filtering rules are further illustrated in "Data sources and data preparation" section.

As illustrated above, approach 1, or the location-conditional development type choice model, first determines the decision-making units set which is the eligible locations through a for loop. And then the NL model is applied to simulate the development choice of each location. The final output would be the spatial distribution of residential development, composed of the individual development decision of each DA. While in approach 2, or the development location choice model, the locations are regarded as the alternatives in the choice set. The formation of choice set is the same ***for*** loop of filtering out the infeasible locations in approach 1. The information of each development event is also required. The second step uses another ***for*** loop to apply the MNL model to each residential project and simulate the probability of choosing each location. The total probability of being chosen at each location represents the “market share” of each location, which determines the spatial distribution of the total residential development.

### Model configuration

The modelling structure of the two approaches both employ random utility formulations. The location choice models have a common concern of spatial correlation. Three types of spatial correlation may affect the model: (1) the spatial correlation among alternatives, (2) the spatial correlation among explanatory variables, and (3) the spatial correlation among the observations. Currently there are mainly two approaches to deal with the spatial correlation in discrete choice modelling, the mixed logit model (ML), and the generalized spatial correlated logit model (GSCLM)^23^. Although spatial correlation is acknowledged in this study, the focus of this paper is the comparison of two different modelling framework of residential development location choice. In the first approach, a nested logit (NL) model is adopted to allow varying correlation among dwelling structure types. In the second approach it is assumed that the utility error terms of all locations are independently and identically distributed, so that a multinomial logit (MNL) model can be applied to the location choice of the development project.

#### Model specification, approach 1

**Figure 3:**
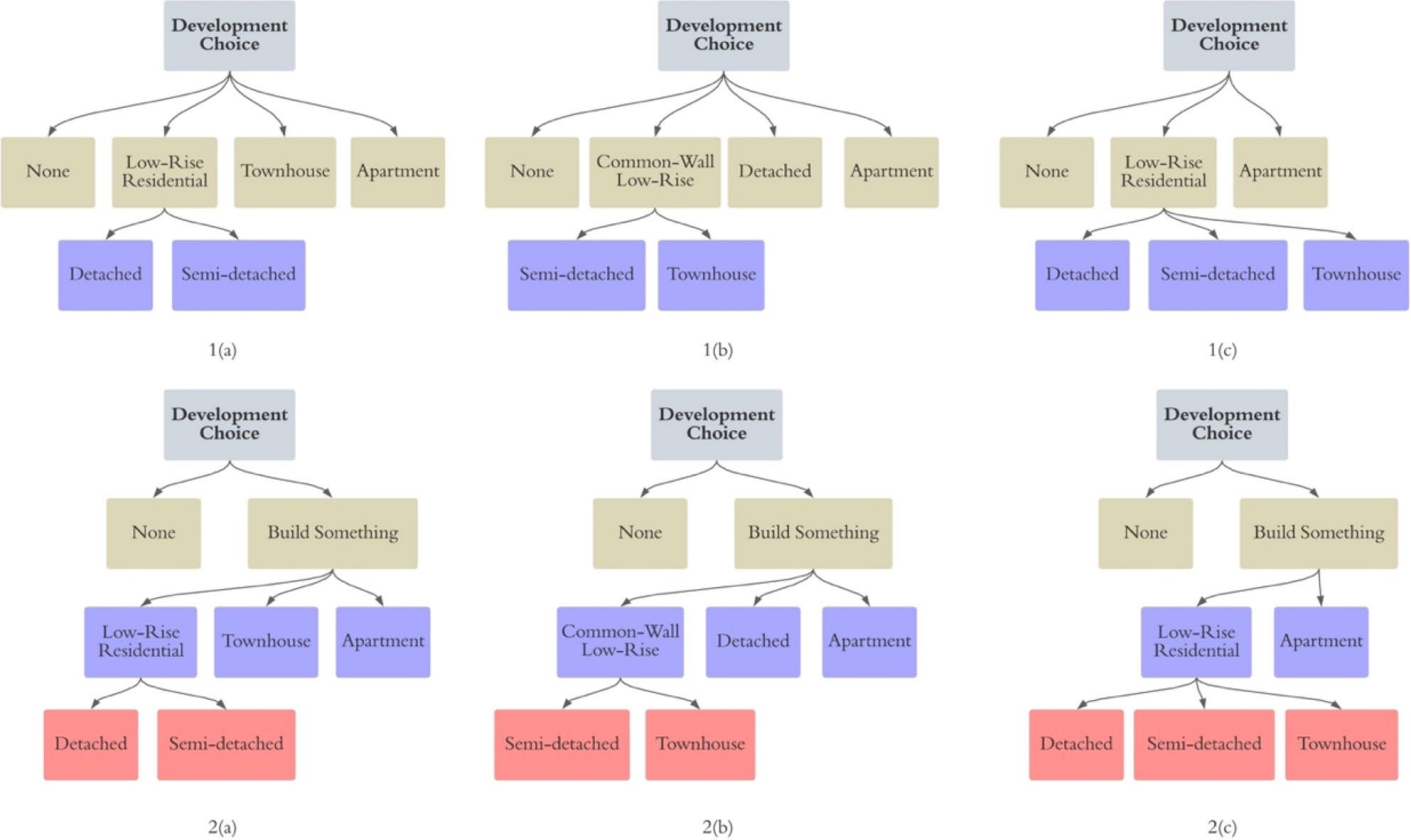
Different nesting structures on the development choice of each location.

In the first approach, a nested logit model is applied to investigate the nested nature of the development decision. Several nesting structures were tested following different classification methods elaborated in Fig. 3. The “None” alternative stands for the choice of not having any residential development, and in the two-layer nesting structure 1(a), 1(b) and 1(c), the none choice is parallel to other development choices, while in 2(a), 2(b), and 2(c), it stands alone at the first layer of a three-layer structure. The classification can have different forms based on the nesting logic. From the data observation and building history perspective, detached and semi-detached housing are classified into the same nest (“Low-rise Residential”), since these two types of dwellings are observed to often be spatially adjacent and built at the same time period, as in the nesting structure (a). Townhouses have only grown in popularity recently, and usually have smaller space, lower privacy and more stories. From a degree of privacy perspective, semi-detached housing and townhouses both have shared walls and thus can be reasonably classified into one nest (“Common-wall Low-Rise”), as in (b). The last classification in (c) is based on the observation that the differences between apartments and the other three dwelling types are wider, in terms of property ownership, heights, etc., thus apartment can stand as one option and the other three dwelling types can be put into the same nest, the “Low-rise Residential”. All six nesting structures were tested. In this analysis, the six nesting structures were found to have very similar goodness-of-fit, while 2(c) was chosen based on the logic that the binary choice of whether or not to have residential development should be the primary decision to make, and that detached, semi-detached and townhouses have more similarities and should be put in the same nest in deciding the structure type . The detailed modelling results of the six structures are presented in Section "Data sources and data preparation".

The probability that the location chooses the development choice $d$ is hierarchically specified in Eq. (1), where the systematic utility function of choosing $d$ in the nest $m$ is in Eq. (2).

1 $$\Pr \left( d \right) = \Pr {(}d{|}m) \times \Pr \left( m \right) = \frac{{e^{{\mu_{d} V_{d|m} }} }}{{\mathop \sum \nolimits_{d^{\prime} \in m} e^{{\mu_{d} V_{d^{\prime}|m} }} }} \times \frac{{e^{{\mu_{m} V_{m} + \frac{{\mu_{m} }}{{\mu_{d} }}ln\mathop \sum \nolimits_{d} \mu_{d} V_{d|m} }} }}{{\mathop \sum \nolimits_{{m^{\prime } \in M}} e^{{\mu_{m} V_{m^{\prime}} + \frac{{\mu_{m} }}{{\mu_{d} }}ln\mathop \sum \nolimits_{d^{\prime}} \mu_{d} V_{{d^{\prime } |m^{\prime } }} }} }}$$

2 $$\left\{ {\begin{array}{*{20}l} {V_{d|m} = \beta_{d|m} + \mathop \sum \limits_{i = 1}^{k} \beta_{i} x_{i,d|m} + \mathop \sum \limits_{j = 1}^{l} \beta_{j,d|m} x_{j} } \hfill \\ {V_{m} = \beta_{m} + \mathop \sum \limits_{{i^{\prime } = 1}}^{{k^{\prime } }} \beta_{i^{\prime}} x_{{i^{\prime } m}} + \mathop \sum \limits_{{j^{\prime } = 1}}^{{l^{\prime } }} \beta_{{j^{\prime } m}} x_{{j^{\prime } }} + V_{d|m} } \hfill \\ \end{array} } \right.$$

${V}_{d|m}$ denotes the lower-level conditional systematic utility function, and ${V}_{m}$ is the upper-level systematic utility function. The expected maximum utility (EMU) of the lower-level choice $d$ for the upper-level choice $m$ is:

$$EMU = \frac{1}{{\mu_{d} }}ln\mathop \sum \limits_{{d^{\prime } \epsilon D}} e^{{\mu_{d} V_{{d^{\prime } |m}} }}$$

#### Model specification, approach 2

**Figure 4:**
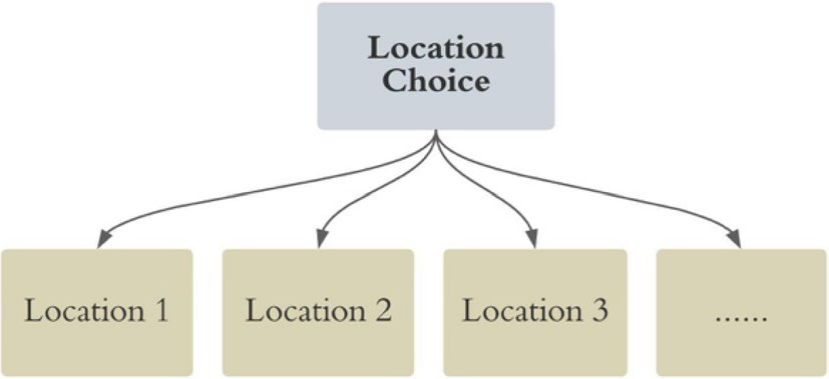
The location choice of residential projects modelling structure.

For the second approach, a multinomial logit model is applied to model the location choice of the residential projects. As shown in Fig. 4, the locations are assumed to be independent and identically distributed alternatives for each residential project.

The probability of choosing one specific location is determined by Eq. (3), where ${V}_{l}$ is the systematic utility linearly functioned as a series of generic and alternative specific variables, as in Eq. (4).

3 $$\Pr \left( l \right) = \frac{{e^{{\mu V_{l} }} }}{{\mathop \sum \nolimits_{{l^{\prime } \epsilon C}} e^{{\mu V_{l^{\prime}} }} }}$$

4 $$V_{l} = \beta_{l} + \mathop \sum \limits_{i = 1}^{m} \beta_{i} x_{il} + \mathop \sum \limits_{j = 1}^{n} \beta_{jl} x_{j} + \epsilon_{l}$$

4 $$V_{l} = \mathop \sum \limits_{i = 1}^{m} \beta_{i} x_{il} + \mathop \sum \limits_{j = 1}^{n} \beta_{j} f\left( {x_{j} ,x_{jl} } \right) + \epsilon_{l}$$

In Eq. (4), ${\beta }_{l}$ is the alternative specific constant for location $l$ , ${x}_{il}$ represents the attributes of the location $l$, the value of which changes across the alternatives, and ${\beta }_{i}$ is the corresponding generic coefficients what do not change across alternatives. The ${x}_{j}$ denotes the attributes of the decision maker, the residential projects, such as the number of dwelling units to be created, the estimated construction cost, which do not change over alternatives, and the ${\beta }_{jl}$ are the alternative-specific coefficients.

## Data sources and data preparation

**Figure 5:**
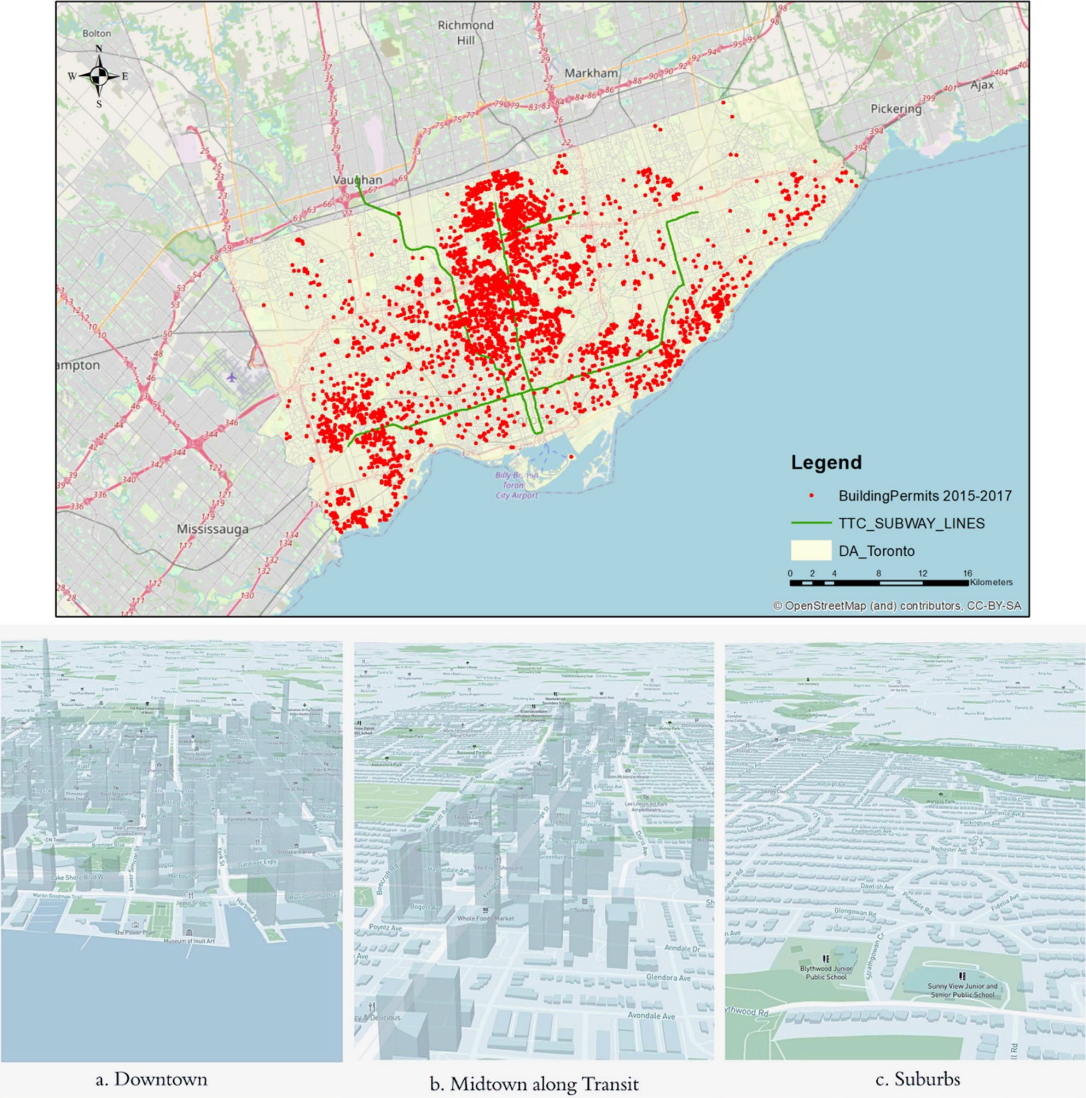
Spatial distribution of the new residential projects in City of Toronto (2015–2017).

The study area is the City of Toronto. The primary data source used is the City’s residential building permits dataset from 2015 to 2017. This dataset is published by City of Toronto Department of Building and Construction. There are 6397 records of cleared residential building permits, of which 2726 are Detached projects, 184 are Semi-detached, 2324 are Townhouses, and 72 are Apartments, as displayed in Supp Fig. 1. The dataset records the location, structure type, date of application and issuance, number of dwelling units to create, estimated construction cost, and land use composition information of each residential project. Figure 5 displays the spatial distribution of the building permit records in the City of Toronto. Many of the projects are located along subway lines, particularly in the midtown and suburbs, where low-rise housing is most prominent.

The datasets employed in this study include:

- Residential Building Permits data of City of Toronto from 2015 to 2017^24^. The point dataset is then associated with the Dissemination Area (DA) shapefile in ArcGIS.
- DA-level spatial boundary data of City of Toronto. The feasible choice set is determined by the land use data and housing attributes in 2016 of each DA. The locations with no available land (categorized as Farmland or Underdeveloped) are removed from the choice set. Those with percentage of water higher than 10%, percentage of park higher than 75%, percentage of residential land lower than 5%, percentage of industrial land higher than 75% are also removed. Those with zero as the average value of dwellings, or zero as the average rent are also removed. Applying these rules resulted in 2434 DAs to include in the feasible choice set for new residential developments.
- 2016 Canadian Census dataset^25^. The census data provides the demographic and socio-economic attributes at the DA level.
- Real Property Solution (RPS) appraisal dataset. The RPS dataset records housing transactions and provides data on average selling prices and number of sales by DA.
- Transportation Toronto Survey (TTS) dataset^26^. The TTS dataset is a comprehensive travel, and road density, percentage of auto as the commuting mode, job accessibility by car, are computed at the DA level.
- DMTI land use dataset. The DMTI land use dataset provides data of the land use composition of each DA.

The variables and data sources are listed in Table 1.

**Table 1:** Variable definitions and data sources.

Variables	Abbreviation	Unit	Source
Price	p	100,000 2016 CAD	RPS property dataset
Number of sales	num	N/A	
Population density	popd	per sq.km	Census 2016
Average household income	hhinc	1,000 2016 CAD	
Road density	roadd	km per sq.km	TTS
Percentage of auto as the primary commuting mode	autop	%	
Job accessibility by car^a^	jacc	N/A	
Log distance to the CBD	dist	log(km)	calculated by the author in ArcGIS
Percentage of parks	ppark	%	DMTI land use dataset
Percentage of open area	popen	%	
Percentage of residential area	pres	%	

^a^Gravity based potential accessibility by car to jobs in all TAZs from the centroid of a TAZ, then interpolated to each DA. The accessibility is calculated based on: ${A}_{i}=\sum_{j=1}^{n}{E}_{j}\times {e}^{-\beta {F}_{ij}}$, where ${E}_{j}$ stands for the number of jobs at zone $j$, ${F}_{ij}$ is the travel time between zone $i$ and zone $j$ by car, and the distance decay parameter $\beta$ was set to 0.05, as the classical first-order estimate of $\beta$ is 1/(average travel time), and the trip-weighted travel time by auto was approximately 20 min during the study period.

## Empirical results of approach 1: modelling the development choice of locations

The six nesting structures in Section "Meta-algorithms of the two approaches" were tested, and the comparison of results can be found in Appendix I in the Supplementary file. The three-layer 2(c) nesting structure, with Building Something or None at the first layer, and Detached, Semi-detached and Townhouse nested in the Low-rise Residential second layer nest, has the best performance, both in terms of the modelling goodness-of-fit and the scale-value of the nest. The coefficient estimation results and the goodness-of-fit indicators for this model are presented in Table 2. The scale value of the two-layer nest, mu-building and mu-lowrise, are estimated as 3.7174 and 2.7855, respectively, indicating that the variation within the nest is small and the nesting structure is effective. The adjusted ${\rho }^{2}$ of the model is 0.8502, which is a is very strong for this type of model.

**Table 2:** Results of the NL model of development choice.

Coefficient	Value	t-test	Coefficient	Value	t-test
Land use parameters			Locational parameters		
b_ppark_sfd	0.003	3.03	b_dist_sfd	− 0.267	− 3.876
b_ppark_sd	0.001	1.213	b_dist_sd	− 0.373	− 3.235
b_ppark_th	− 0.003	− 1.764	b_dist_th	− 0.263	− 3.346
b_ppark_apt	0.001	2.724	b_dist_apt	− 0.207	− 1.896
b_popen_sfd	− 0.083	− 3.047	b_jacc_sfd	1.707	5.039
b_popen_sd	− 0.07	− 2.721	b_jacc_sd	1.68	3.044
b_popen_th	− 0.076	− 2.832	b_jacc_th	2.03	3.625
b_popen_apt	− 0.067	− 2.61	b_jacc_apt	2.12	4.148
b_pres_sfd	0.007	5.16	b_cost_sfd	0.05	2.252
b_pres_sd	0.008	2.341	b_cost_sd	− 0.424	− 5.21
b_pres_th	− 0.003	− 2.321	b_cost_th	− 0.424	− 5.263
b_pres_apt	− 0.002	− 2.966	b_cost_apt	− 0.119	− 3.047

Demographic parameters			Density parameters		
b_autop_sfd	1.214	2.637	b_popd_sfd	− 0.116	− 6.693
b_autop_sd	1.411	4.302	b_popd_sd	− 0.108	− 3.745
b_autop_th	0.649	2.206	b_popd_th	− 0.073	− 4.235
b_autop_apt	0.568	2.252	b_popd_apt	− 0.075	− 3.963
b_hhinc_sfd	0.026	4.239	b_roadd_sfd	− 0.007	− 4.601
b_hhinc_sd	0.03	5.263	b_roadd_sd	0.003	2.351
b_hhinc_th	0.031	3.757	b_roadd_th	− 0.004	− 2.429
b_hhinc_apt	0.026	2.074	b_roadd_apt	0.001	− 3.371
b_value_sfd	0.103	3.473	b_p	0.407	3.893
b_value_sd	0.122	3.215	b_numsale	0.002	5.02
b_value_th	0.454	1.662	**mu_lowrise**	**2.786**	**15.588**
b_value_apt	0.538	1.833	**mu_build**	**3.717**	**5.678**
			**LL(0)**	− **6077.24**	
			**LL(final)**	− **858.15**	
			**Adj.** ${\rho }^{2}$^a^	**0.8502**	

Significant values are in bold.

^a^Calculation of rho-squared is ${\rho }^{2}=1-\frac{{LL}^{*}(\beta )}{{LL}^{*}(0)}$ , and adjusted rho-squared is $adj.{\rho }^{2}=1-\frac{{LL}^{*}(\beta )}{{LL}^{*}(0)}\times \frac{n}{n-B}$ . Adjusted ${\rho }^{2}$ of value of 0.2–0.3 is considered reasonable.

Number of sales and average sales price of each structure type are found to be statistically significant with positive coefficients. The factors included in the model are mostly significant. The percentage of parks has significant positive coefficient for single family detached housing projects, but not for other dwelling types. The presence of parks is generally an attractive attribute for residential neighbourhoods. Nevertheless, the percentage of park in the neighbourhood does not measure the distance to the entrance or percentage of dwellings having a park view, and the utility added from a pleasant natural environment can be limited. The coefficient of this variable is not expected to be always positive. The percentage of open area has significant negative coefficients for all dwelling types. The percentage of residential land has positive coefficients for single detached and semi-detached houses, but negative coefficients for townhouse and apartment buildings. The lower-rise residential projects need large area, thus DAs with higher percentage of residential land would be preferred. While for apartment buildings, DAs with mixed land use would be preferred.

The distance from city centre has a significant negative influence for all residential development. However, the percentage of autos as the primary commuting mode and job accessibility by car have significant positive coefficients. These indicate that although the central area is overall more attractive for residential development, accessibility plays a key role, and the auto-oriented suburbs with good access to jobs are also preferred. In terms of the demographic factors, neighbourhoods with higher average household income, higher average dwelling values, are more likely to have new housing development. The population density and road density both negatively influence the potential for new housing development, due to the limited land availability in the densely build areas.

Table 3 reports the aggregate direct and cross elasticity of each development choice with respect to each variable. The direct elasticity reflects how the probability of the alternative changes as the value of a variable changes in the alternative’s utility function, while the cross elasticity reflects how the probability of an alternative’s choice changing as the value of the variable changes for other alternatives. The direct and cross elasticities can be used to interpret the level of influence of each variable on the choice probabilities. From Table 3, most elasticities have absolute value smaller than 1, except for semi-detached housing with respect to log distance to the CBD, townhouses with respect to percentage of residential area, and apartments with respect to job accessibility by car. This indicates that the probability of choosing each alternative, or the residential housing supply, are not elastic to most variables. The probability of choosing to build the detached structure type increases approximately 0.15% in response to a one percent change in the detached unit sales price, while the percent changes for semi-detached, townhouses, and apartment are 0.61, 0.56 and 0.26, respectively. Elasticities with respect to the number of sales are relatively low, except for apartments, which have a higher value. The same interpretation can be applied for other variables. All the alternatives are inelastic to population density, average dwelling value, average household income, road density, percentage of park, percentage of open area. This is an interesting result in that these are all attributes that can be expected to be positively correlated with household location choice, in other words, the factors influencing housing development decisions are not necessarily the same as those that are valued by households. The cross elasticities were examined for only the price of the alternatives, and were found to all be negative and inelastic.

**Table 3:** Direct and cross elasticity of the NL model^a^.

	SFD	SD	TH	APT
Aggregate direct elasticity				
Price	0.147	0.614	0.562	0.263
Number of sales	0.011	0.016	0.020	0.199
Population density	− 0.090	− 0.050	0.353	0.178
Log distance to the CBD	0.031	2.700	0.451	− 0.842
Average dwelling value	− 0.056	− 0.099	0.477	0.262
Average household income	− 0.003	0.147	0.128	0.069
Road density	− 0.044	− 0.815	0.022	0.117
Auto percentage	0.049	0.903	− 0.334	− 0.352
Percentage of park	0.009	− 0.014	− 0.079	− 0.026
Percentage of open area	− 0.012	0.058	− 0.006	0.071
Percentage of residential area	0.137	0.505	− 1.150	− 0.661
Job accessibility by car	− 0.017	0.011	0.911	1.210
Aggregate cross elasticity				
SFD to price of TH	− 0.019			
TH to price of SFD			− 0.601	
TH to price of APT			− 0.038	

^a^SFD, SD, TH, APT refers to single family detached, semi-detached, townhouse, apartment, respectively.

The model validation was conducted by predicting the development choice on 10 bootstrapped samples. And the validation results are included in the Supplementary file. Applying this analysis framework, the share of each structure type, and the percentage of locations that start no residential development, can be well predicted. However, the model cannot accurately predict the choice of an individual DA. The assumption that the three-layer nesting structure can separate the “None” choice from “Doing something” and should have better performance than other nesting structures is supported by the empirical study, and the scale parameters of this nesting structure are effective. The estimation of coefficients and elasticity analysis of the variates indicate that while the average sold number and price of each dwelling type significantly influence the decision-making of each location, the properties of the location itself determine which residential type will be developed into.

## Empirical results of approach 2: modelling the location choice of residential projects

The second approach models the location choice of residential projects, which involves the discrete location choice for each project from a large number of potential alternatives. A rich literature exists on how to deal with large spatial choice sets, starting from McFadden^27^ when he first structured the discrete choice modelling of residential location choice. He demonstrated that the most disaggregated level of modelling will require infeasible levels of data processing, as well as violates the logit model’s IIA assumption. He further showed that a random/fixed sample can be used to estimate the parameters if the function form is valid, which would be consistent with the full choice set. He then introduced three sampling approaches, the first one to be a fixed subset of the full set independent of the observed choice, the second to be a random subset plus the chosen alternative, and the third one consisting of a subset with the chosen alternative and one or more randomly selected alternatives from other partitioned subsets. Lee and Waddell^18^ used random sampling of alternatives in the residential mobility and location choice modelling, while introducing a correction term for sampling bias. These sampling approaches do not consider the choice set formation process of the decision makers, and cannot replicate the real choice set.

Another stream of choice set specification research applies importance sampling. Importance samples are typically stratified: alternatives most likely to be chosen are sampled at a higher rate, followed by alternatives with a priori lower choice probabilities, for a number of strata defined by the researchers. Li et al.^28^ developed the approach of geographical stratified importance sampling, which assigns different selection probability to alternatives in different strata, and the boundaries of the stratum regions are determined when there was no spatial dependency within the stratum to ensure homogeneity, and Moran’s I statistic is used to examine the clustering pattern. In Bowman and Ben-Akiva^29^’s modelling of residential location choice, zones located within a central city area and in the same income bracket as decision makers’ current home zones were preferentially sampled. Distance based importance sampling is also commonly used^30,31^. Curtis‐Ham et al.^32^ oversample potential crime locations near to offenders’ activity locations that are more likely to be chosen.

The above sampling methods all estimate the parameters and make the predictions on the full choice set, with an artificially formed subset, which is problematic per se as the these are likely to be far from the overlap between the decision-maker’s actual awareness and feasible sets. This study first examined two types of constraints using the land use data set and property transaction dataset as the filters to form the feasible choice set. However, the constraints are still arbitrary and would result in the removal of the chosen alternatives. Thus, this study keeps the universal choice set in the empirical analysis (The constraints examined include: 1) remove the alternative DAs with percentage of water body higher than 90%, or percentage of residential land lower than 5%, or percentage of parks higher than 90%, using the DMTI land use dataset; 2) remove the alternative DAs with no transaction records in the RPS property transaction database from 2015 to 2017. Though this study applied the universal set in the empirical analysis, the authors still propose that a registered land for sell dataset, if available, should have been used to limit the number of alternatives involved.).

The data and variables in this approach use the same variables listed in Table 1 except that approach 2 has three variables representing attributes of the residential project: the structure type, estimated construction cost and number of dwellings to be created. The data of the project-specific variables are applied from the building permits dataset. The project attributes are added into the model interacting with location variables (since the project attributes do not vary across locations for a given project). The dummy variables of structure type are interacted with the percentage of auto as the primary commute mode in the DA, the road density and the job accessibility by car. The estimated construction cost and number of dwelling units to be created interact with the average sales price of the dwelling units in the DA: the average sales price of the dwelling units is multiplied by the number of dwelling units to be created and divided by the estimated construction cost, to represent the relative profitability for the project to choose the DA. An MNL model is employed, and the estimation results of the variables are presented in Table 4.

**Table 4:** Results of the MNL model of location choice.

Coefficient	Valuer	t-test	Coefficient	Value	t-test
b_pres	− 0.011	− 20.817	b_autop	1.947	12.267
b_ppark	− 0.008	− 8.349	b_sd_autop	− 2.254	− 4.028
b_popen	0.037	27.370	b_apt_autop	− 5.880	− 4.591
b_roaddens	− 0.042	− 12.319	b_th_autop	− 3.798	− 17.900
b_sd_roadd	0.087	13.586	b_jacc	0.293	3.808
b_apt_roadd	0.065	5.711	b_sd_jacc	− 1.596	− 5.221
b_th_roadd	0.032	7.418	b_apt_jacc	0.440	0.754
b_popdens	1.096	0.241	b_th_jacc	− 0.060	− 0.513
b_hhinc	0.004	2.745	b_num	− 0.711	− 6.550
b_val	0.937	14.187	LL(0)		− 39,964.37
b_dist	0.291	6.753	LL(final)		− 37,595.09
b_price	− 4.723	− 33.141	Adj.${{\varvec{\rho}}}^{2}$		0.0588

All the coefficients are statistically significant, although the adjusted ${\rho }^{2}$ is only around 0.059, which is understandable in that predicting one specific location in a large choice set is difficult. The coefficient signs are consistent with the results of approach 1. The interaction variable of average housing sale price and the project construction cost and number of dwelling units shows a significant negative influence on the probability to be chosen, indicating that projects with high construction cost per dwelling unit, usually the detached unit projects, tend to locate in areas with lower housing values, as the high-value area involves even higher land cost. Projects with different structure type have different coefficients for the road density and percentage of auto as the primary commuting mode when choosing the location. High road density has a positive influence on semi-detached and apartment projects, while single detached and town house projects have negative parameters. Detached housing projects are associated with areas with high auto percentage in their commuting mode, while apartment projects negatively corelate with this variable in their choice. Job accessibility by car positively influences the probability to be chosen by detached housing. In general, areas with higher dwelling value, and more open area, are more likely to be chosen by the residential project.

**Figure 6:**
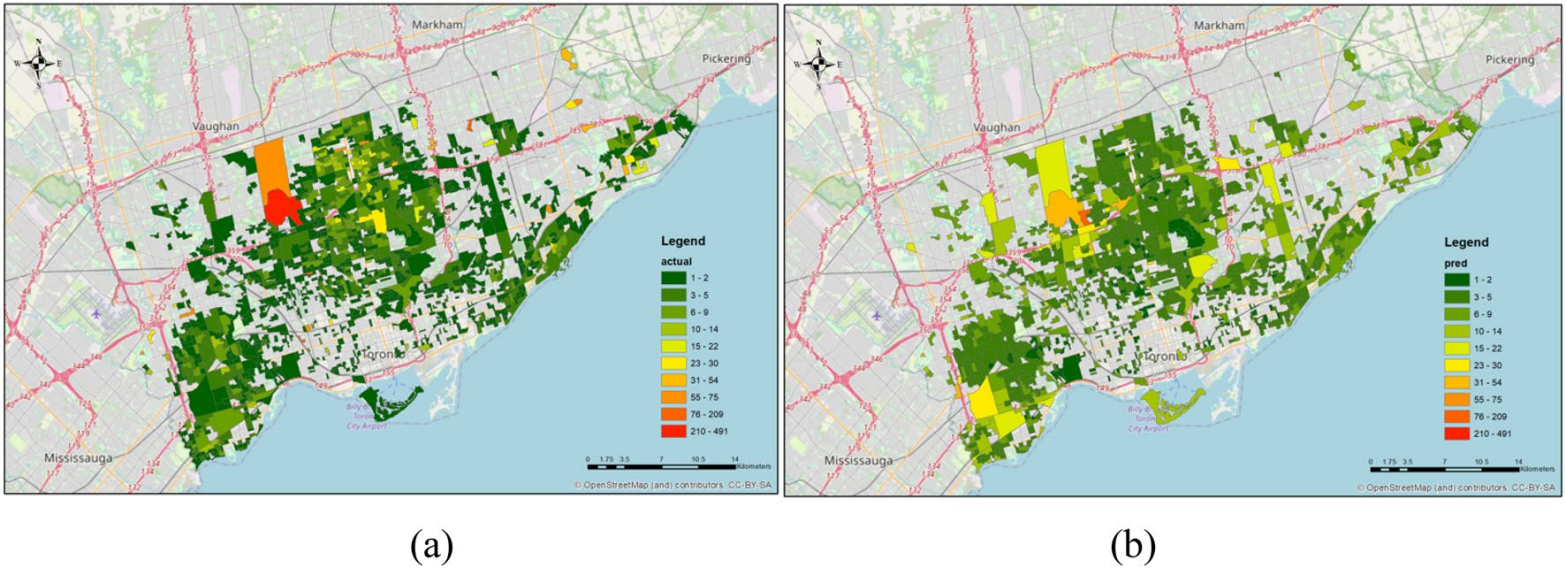
Actual (**a**) and Predicted (**b**) location choice of new residential projects in City of Toronto applying approach 2.

With the estimated parameters, the model predicts the location choice of each residential project. Most of the DAs are predicted with probabilities close to zero, even for the accurately predicted alternative. The model only accurately predicts 36 out of 5636 project location choices, although the mean absolute error (The mean absolute error is calculated as: $MAE=\frac{\sum_{i=1}^{n}\left|{y}_{i}-{x}_{i}\right|}{n}$, where ${y}_{i}$ denotes the predicted probability, ${x}_{i}$ denotes the actual probability (0 or 1), and n is the number of cases (number of decision maker times the number of alternatives).) of the prediction is 0.00166. For each location alternative, the average difference between the predicted and actual frequency being chosen is 4.61. These results indicate that predicting the individual location choice of each development project is still challenging with large choice set and limited information on the available land and site selection preference. The predicted and observed spatial distributions of residential housing projects are in shown in Fig. 6. Overall, the prediction captures the spatial distribution pattern of the new residential projects. I.e., while, the correct prediction of the location of an individual housing project is difficult, the model does a reasonable job of predicting, in the aggregate, the spatial distribution of new housing starts. The prediction distributes the residential projects more evenly over space, while the actual distribution is more clustered, especially within the north suburban and midtown areas along the Yonge subway line.

A section of the comparison of the two approaches in data requirement, estimation process, prediction performance and function in the urban simulation systems are detailed in the Supplementary file.

## Comparison of the two approaches

### Data requirements

Modelling the housing development choice of each location (approach 1), and the location choice of each residential project (approach 2) both require considerable data regarding the attributes of the location. However, the specific features of residential projects such as project size and construction cost are not included in the explanatory variables for approach 1. Approach 2 does not directly include the attributes of residential projects either, because location choice modelling involves a large number of alternatives, and the parameters specific to each of the alternatives are not of interest in this application. By interacting with the DA variables, we were able to incorporate variables such as estimated construction cost, structure type, and number of dwelling units created in approach 2. The attributes of the actual developers will affect the location choice in approach 2, however, these are not included as generic variables due to limited available data. The “choice results” are based on the construction starting date and location of residential projects for the model time period. Thus, the data requirements for the two approaches are not drastically different, except that approach 2 requires attributes of construction projects.

### Number of parameters and estimation

Approach 1 models the development choice among no development, single family detached housing, semi-detached housing, townhouses, and apartment. The variables included in the model are mostly attributes of the “decision-maker”, or the location itself, which hold constant across alternatives, and thus approach 1 requires more alternative specific coefficients. The estimation is much less computationally intensive with fewer alternatives. While for approach 2, the modelling of location choice means large numbers of alternatives, and most of the variables included in the model vary across alternatives. The parameters can hold constant across alternatives, except for the variables that interact with the attributes of the development projects. This study includes the residential project features through interaction terms. The estimation running time of approach 1 is much less than approach 2 (approach 1 takes one minute 28 s, and approach 2 takes 1 h 10 min 55 s on a laptop with 8 GB RAM).

### Prediction results

Although with lower ${\rho }^{2}$, approach 2 works well in the aggregate predictions of what type of housing gets developed where, and has better performance than the goodness of fit indicates. Challenges exist in identifying the exact location of each residential project at the DA level, but the estimated parameters can help to understand and explain the locational preference of different residential projects. Approach 1 has good prediction performance in replicating the market share of each dwelling type in the city, while approach 2 works better in explaining the locational preference of individual housing developments. In terms of prediction sensitivity, the model prediction in approach 1 relies highly on the market share of the empirical dataset; approach 2 is sensitive to the number of alternatives in the choice set, and all alternatives are predicted with low probability with a large number of alternatives. This could be improved by applying a constrained choice set formed with land sell database, and including more features of the housing projects and builders.

### Function in an urban simulation system

In an urban microsimulation system, the housing supply module needs a model component that predicts the total number of dwellings to be constructed at a given time step. The spatial distribution model then allocates the total supply to different locations. Approach 1 models the development choice of each location, determining its probability of being converted into a different type of dwelling or remaining undeveloped. While the parameters applied in approach 1 are based on empirical estimations, it fits well into the housing supply model and identifies the dwelling type and location. The total number of dwellings to be allocated to each location, however, remains unsolved by approach 1.

The discrete choice model in approach 2 tends to serve as a ranking system which should effectively identify the most probable locations for one specific project, based on both the features of the project, and the features of the location. Due to the limitation in available data of the choice set and the attributes of housing projects, approach 2 did not work well in predicting the location choice of individual housing project in the empirical study, but it can predict the overall spatial distribution of the residential projects. When distributing the total housing stock in an urban microsimulation model system, approach 2 is capable of determining the location of housing projects of different structure type and size. Additional data of the features of residential projects and the choice set are needed for approach 2 to be applied in an urban simulation system.

## Discussion

This study discusses two approaches for modelling the spatial distribution of new housing supply: the first approach models the development choice of each location; the second approach models the location choice of each residential development project. Modelling frameworks for the two approaches are elaborated in terms of choice set formation, spatial scale determination, and decision-making unit determination. The utility function of the two approaches specifies project characteristics such as structure type and construction cost, as well as location characteristics such as housing price, number of sales, and population density. Multinominal logit and nested logit models are applied to empirical data in the City of Toronto. The estimation and prediction results of the two approaches are compared.

Both approaches have pros and cons when it comes to estimation, prediction, and application within an urban microsimulation system. The study has two limitations. First, the model does not include variables related to the features of the developers due to limited data availability. A more complete dataset should be used to conduct further modelling. Second, neither of the two approaches is capable of predicting the location and the number of dwelling units of the new housing supply simultaneously. Results should also be examined using other models, such as the MDCEV model, which is the focus of further work by the authors.

### Supplementary Information

Supplementary Information.

## Data Availability

Some data and models that support the findings of this study are available from the corresponding author upon reasonable request. Some data used during the study are proprietary or confidential in nature and may only be provided with restrictions.
