# Supplementary material for: Analyzing housing supply location choice: a comparative study of the modelling frameworks
Source: Sci Rep. 2024 Jan 16;14:1435. doi: 10.1038/s41598-024-51754-9 (PMC10791635; doi:10.1038/s41598-024-51754-9)
Supplement: Supplementary file 1 — Supplementary Information. [file 41598_2024_51754_MOESM1_ESM.docx]

**Supplementary File 1**


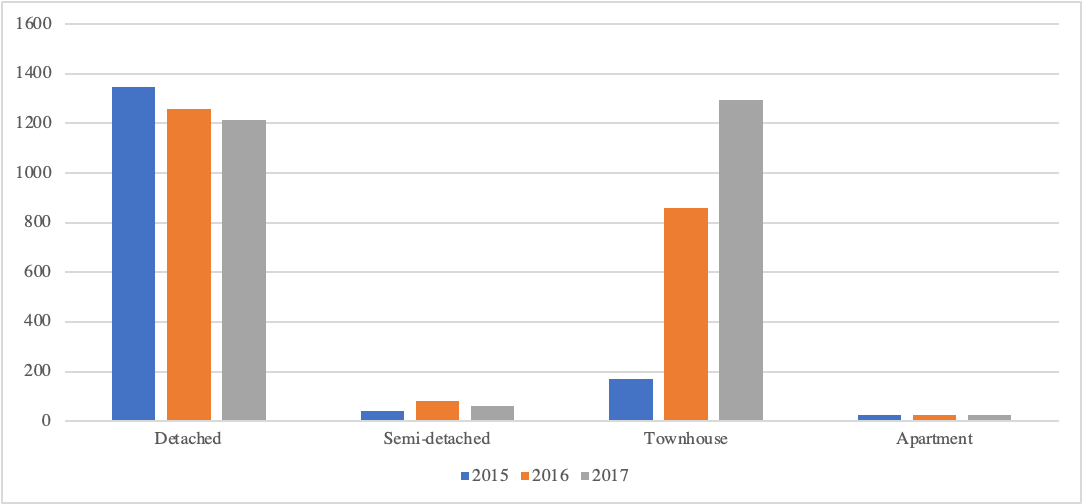


Supp Fig1. Residential building permit records by year by structure type

**Supplementary File 2 Model Validation on Bootstrapped Samples**

Supp Tab1 and Supp Tab2 report model predicted shares of each development choice, the real share in the sample, and the summary of the prediction performance of the model on the 10 bootstrap samples. For each bootstrap sample, the market share is distorted slightly from the original share, but the model is capable of replicating the share of each development choice in general. The model has a slight tendency to underestimate the probability of choosing the none and detached alternatives, while overpredicting the semi-detached and apartment choice, which should be modified in operational application by further adjustment in the model formulation or by including enhanced explanatory variables related to the development of semi-detached housing.

Supp Tab1. Results of 10 bootstrap samples: observed and predicted market share

|  | B1_real | B1_sim | B2_real | B2_sim | B3_real | B3_sim | B4_real | B4_sim | B5_real | B5_sim |
| --- | --- | --- | --- | --- | --- | --- | --- | --- | --- | --- |
| None | 63.14% | 63.70% | 63.32% | 62.23% | 63.90% | 60.88% | 64.99% | 63.20% | 64.12% | 62.26% |
| SFD | 31.30% | 25.16% | 30.64% | 27.71% | 30.77% | 30.16% | 29.93% | 27.46% | 30.85% | 28.07% |
| SD | 1.75% | 5.49% | 2.07% | 5.66% | 1.72% | 5.36% | 1.67% | 4.96% | 1.77% | 5.56% |
| TH | 2.01% | 2.20% | 1.96% | 2.25% | 1.75% | 0.90% | 1.64% | 1.57% | 1.91% | 1.58% |
| APT | 1.80% | 3.44% | 2.01% | 2.06% | 1.85% | 2.69% | 1.77% | 2.91% | 1.35% | 2.64% |
|  | B6_real | B6_sim | B7_real | B7_sim | B8_real | B8_sim | B9_real | B9_sim | B10_real | B10_sim |
| None | 64.49% | 64.59% | 62.66% | 62.11% | 64.22% | 62.60% | 63.90% | 62.54% | 64.38% | 62.35% |
| SFD | 30.35% | 26.59% | 32.02% | 27.20% | 30.67% | 27.81% | 31.44% | 27.57% | 30.32% | 26.00% |
| SD | 1.75% | 3.16% | 1.69% | 5.69% | 1.83% | 5.29% | 1.35% | 4.79% | 1.64% | 5.46% |
| TH | 2.17% | 2.58% | 2.01% | 2.23% | 1.69% | 1.84% | 1.91% | 1.97% | 2.09% | 2.45% |
| APT | 1.24% | 3.17% | 1.62% | 2.66% | 1.59% | 2.45% | 1.40% | 3.23% | 1.56% | 3.74% |

Supp Tab2. Validation by 10 bootstrap sample simulation

|  | Mean Prediction Error | Max Under Prediction | Max Over Prediction |
| --- | --- | --- | --- |
| None | -1.27% | -3.02% | 0.56% |
| SFD | -3.46% | -6.14% | -0.61% |
| SD | 3.42% | 1.41% | 3.99% |
| TH | 0.04% | -0.85% | 0.41% |
| APT | 1.28% | 0.05% | 2.18% |

Supp Tab3. Comparison of the two approaches

|  |  | Approach 1: Modelling the Development Choice of the Location | Approach 2: Modelling the Location Choice of Residential Projects |
| --- | --- | --- | --- |
| Data Requirement | locational characteristics | Generic variables are all included.  Alternative specific variables, such as sale price for specific dwelling type at this location, are included. | Alternative specific variables are all included. |
|  | features of the residential project | Not required. The development choice is made based on the features of the location, and attributes of each alternative, the structure type. | Features of each residential project such as estimated construction cost and structure type are required.  The variables representing the project features are interacted with the variables of each alternative to be included in the model. |
|  | features of the developer | N/A | Not applied due to data availability. |
| Parameters & Estimation | number of parameters | Limited number of alternative specific parameters and constant parameters. | Interacting the generic variables with the alternative specific variables intensively reduces the number of parameters. |
|  | estimation | Fast and effective estimation process.  Higher goodness-of-fit ($\rho^{2}$) of the model could be achieved. | Burdensome estimation process with massive number of parameters.  Very low goodness-of-fit ($\rho^{2}$) of the model expected. |
| Prediction | prediction accuracy | Higher accuracy in identifying the total market share of each alternative.  Not good at distributing the dwelling units over space. | Lower accuracy for individual housing project.  Works well in predicting the spatial distribution of projects of different structure type and size. |
|  | sensitivity | Sensitive to the market share of each alternative in the empirical dataset. | The number of alternatives affect the predicted probability. |
| Function in an urban microsimulation system | decision-making unit | The location. | The residential project. |
|  | treatment of developers | No developers involved. | Developers’ attributes could influence the location choice of the project. |
|  | pre-requisites | Need to pre-determine the total number of dwelling units. | Need to pre-determine the total number of dwelling units of each dwelling type from each developer. |
|  | product | The individual location’s development choice; the market share of each alternative dwelling type. | The location choice of each residential project. |

**Supplementary File 4**

Appendix I: Results of multiple nesting structure in the NL model of development choice

|  | 1a | | 1b | | 1c | | 2a | | 2b | | 2c | |
| --- | --- | --- | --- | --- | --- | --- | --- | --- | --- | --- | --- | --- |
|  | Value | t-test | Value | t-test | Value | t-test | Value | t-test | Value | t-test | Value | t-test |
| asc_sfd | -3.378 | -0.076 | -3.468 | -0.325 | -3.313 | -0.185 | -3.251 | -2.246 | -3.269 | -1.187 | -3.314 | -3.697 |
| asc_sd | -3.599 | -0.178 | -3.276 | -0.507 | -3.526 | -0.305 | -3.333 | -1.557 | -3.287 | -0.564 | -3.380 | -2.572 |
| asc_th | -4.007 | -0.274 | -3.789 | -0.862 | -3.869 | -0.009 | -3.506 | -5.573 | -3.465 | -0.507 | -3.502 | -3.755 |
| asc_apt | -6.138 | -1.074 | -6.131 | -0.440 | -6.124 | -0.104 | -4.140 | -1.654 | -4.163 | -0.900 | -3.974 | -3.595 |
| b_ppark_sfd | 0.010 | 0.197 | 0.010 | 0.219 | 0.009 | 0.014 | 0.003 | 1.193 | 0.003 | 0.376 | 0.003 | 3.030 |
| b_ppark_sd | 0.006 | 0.129 | 0.002 | 0.120 | 0.005 | 0.029 | 0.002 | 0.705 | 0.001 | 0.495 | 0.001 | 1.213 |
| b_ppark_th | -0.007 | -0.150 | -0.006 | -0.141 | -0.005 | -0.010 | -0.002 | -2.620 | -0.002 | -0.700 | -0.003 | -1.764 |
| b_ppark_apt | -0.001 | -1.190 | -0.001 | -0.024 | -0.001 | -0.032 | 0.000 | -0.253 | 0.000 | -0.670 | 0.001 | 2.724 |
| b_popen_sfd | -0.107 | -0.281 | -0.108 | -0.673 | -0.106 | -0.204 | -0.083 | -1.569 | -0.083 | -1.236 | -0.083 | -3.047 |
| b_popen_sd | -0.079 | -0.165 | -0.076 | -0.977 | -0.077 | -0.158 | -0.073 | -1.365 | -0.072 | -2.400 | -0.070 | -2.721 |
| b_popen_th | -0.088 | -0.309 | -0.087 | -1.081 | -0.089 | -0.027 | -0.076 | -1.366 | -0.076 | -1.025 | -0.076 | -2.832 |
| b_popen_apt | -0.055 | -0.245 | -0.056 | -1.083 | -0.055 | -0.042 | -0.066 | -1.139 | -0.066 | -0.918 | -0.067 | -2.610 |
| b_pres_sfd | 0.020 | 0.988 | 0.020 | 4.716 | 0.019 | 0.053 | 0.007 | 1.200 | 0.007 | 0.695 | 0.007 | 5.160 |
| b_pres_sd | 0.023 | 2.813 | 0.018 | 0.149 | 0.021 | 0.037 | 0.008 | 0.736 | 0.007 | 0.401 | 0.008 | 2.341 |
| b_pres_th | -0.009 | -0.330 | -0.006 | -1.070 | -0.005 | -0.006 | -0.002 | -1.145 | -0.001 | -0.833 | -0.003 | -2.321 |
| b_pres_apt | -0.007 | -0.330 | -0.007 | -0.128 | -0.007 | -0.105 | -0.002 | -1.109 | -0.002 | -0.428 | -0.002 | -2.966 |
| b_roadd_sfd | -0.014 | -0.588 | -0.017 | -0.169 | -0.014 | -0.098 | -0.007 | -1.916 | -0.007 | -0.340 | -0.007 | -4.601 |
| b_roadd_sd | 0.009 | 1.010 | 0.010 | 0.204 | 0.011 | 0.028 | 0.001 | 1.933 | 0.001 | 0.373 | 0.003 | 2.351 |
| b_roadd_th | -0.006 | -0.271 | -0.001 | -0.072 | -0.007 | -0.026 | -0.004 | -1.051 | -0.004 | -2.524 | -0.004 | -2.429 |
| b_roadd_apt | 0.002 | 0.038 | 0.002 | 0.040 | 0.002 | 0.019 | 0.000 | -0.643 | 0.000 | -1.079 | 0.001 | -3.371 |
| b_jacc_sfd | 1.122 | 1.282 | 1.125 | 0.628 | 1.163 | 0.032 | 1.717 | 1.834 | 1.713 | 0.553 | 1.707 | 5.039 |
| b_jacc_sd | 1.063 | 0.339 | 1.171 | 0.306 | 1.129 | 0.030 | 1.678 | 2.190 | 1.685 | 1.657 | 1.680 | 3.044 |
| b_jacc_th | 2.085 | 0.299 | 2.008 | 1.292 | 1.928 | 0.010 | 1.991 | 1.699 | 1.980 | 0.319 | 2.030 | 3.625 |
| b_jacc_apt | 2.708 | 0.300 | 2.725 | 0.154 | 2.728 | 0.125 | 2.212 | 1.125 | 2.225 | 0.478 | 2.120 | 4.148 |
| b_popd_sfd | -0.188 | -0.486 | -0.188 | -0.852 | -0.182 | -0.383 | -0.115 | -1.766 | -0.115 | -0.776 | -0.116 | -6.693 |
| b_popd_sd | -0.173 | -0.848 | -0.151 | -0.197 | -0.164 | -0.083 | -0.110 | -1.290 | -0.106 | -0.400 | -0.108 | -3.745 |
| b_popd_th | -0.069 | -0.550 | -0.076 | -0.313 | -0.079 | -0.039 | -0.076 | -1.579 | -0.077 | -0.716 | -0.073 | -4.235 |
| b_popd_apt | -0.059 | -0.428 | -0.060 | -0.418 | -0.060 | -0.024 | -0.074 | -1.493 | -0.074 | -0.647 | -0.075 | -3.963 |
| b_hhinc_sfd | 0.015 | 0.160 | 0.015 | 1.354 | 0.016 | 0.050 | 0.026 | 1.607 | 0.026 | 4.371 | 0.026 | 4.239 |
| b_hhinc_sd | 0.025 | 0.084 | 0.027 | 0.330 | 0.027 | 0.697 | 0.030 | 2.185 | 0.030 | 1.843 | 0.030 | 5.263 |
| b_hhinc_th | 0.025 | 0.384 | 0.026 | 0.962 | 0.024 | 0.019 | 0.030 | 1.335 | 0.030 | 1.504 | 0.031 | 3.757 |
| b_hhinc_apt | 0.019 | 0.042 | 0.020 | 0.031 | 0.019 | 0.371 | 0.026 | 0.459 | 0.026 | 0.658 | 0.026 | 2.074 |
| b_value_sfd | -0.456 | -0.545 | -0.418 | -0.146 | -0.400 | -1.065 | 0.160 | 1.242 | 0.165 | 0.180 | 0.103 | 3.473 |
| b_value_sd | -0.393 | -0.105 | -0.379 | -0.197 | -0.395 | -0.132 | 0.201 | 0.647 | 0.211 | 0.923 | 0.122 | 3.215 |
| b_value_th | 0.402 | 0.457 | 0.295 | 0.445 | 0.398 | 0.011 | 0.488 | 1.348 | 0.471 | 0.264 | 0.454 | 1.662 |
| b_value_apt | 0.324 | 0.048 | 0.322 | 0.203 | 0.334 | 0.035 | 0.553 | 0.972 | 0.553 | 0.535 | 0.538 | 1.833 |
| b_dist_sfd | -0.309 | -0.076 | -0.297 | -0.550 | -0.309 | -0.093 | -0.274 | -2.385 | -0.272 | -3.098 | -0.267 | -3.876 |
| b_dist_sd | -0.534 | -0.251 | -0.561 | -1.929 | -0.549 | -0.094 | -0.353 | -1.468 | -0.356 | -0.425 | -0.373 | -3.235 |
| b_dist_th | -0.293 | -0.147 | -0.321 | -0.655 | -0.294 | -0.007 | -0.262 | -2.188 | -0.267 | -1.430 | -0.263 | -3.346 |
| b_dist_apt | -0.079 | -0.668 | -0.080 | -0.322 | -0.079 | -0.014 | -0.196 | -1.068 | -0.195 | -1.009 | -0.207 | -1.896 |
| b_cost_sfd | -0.294 | 9.455 | -0.295 | 2.880 | -0.293 | 3.463 | -0.033 | 1.587 | -0.058 | 3.026 | 0.050 | 2.252 |
| b_cost_sd | -0.294 | 9.534 | -0.296 | 2.899 | -0.294 | 3.493 | -0.034 | 1.672 | -0.059 | 2.727 | -0.424 | -5.210 |
| b_cost_th | -0.297 | 9.301 | -0.298 | 2.885 | -0.296 | 3.474 | -0.034 | 1.515 | -0.060 | 3.118 | -0.424 | -5.263 |
| b_cost_apt | -0.301 | 8.890 | -0.302 | 2.877 | -0.301 | 3.511 | -0.036 | 1.429 | -0.061 | 2.796 | -0.119 | -3.047 |
| b_autop_sfd | 1.401 | 0.232 | 1.393 | 0.216 | 1.360 | 0.615 | 1.196 | 2.717 | 1.198 | 0.965 | 1.214 | 2.637 |
| b_autop_sd | 1.981 | 0.455 | 1.600 | 0.171 | 1.820 | 0.041 | 1.401 | 1.366 | 1.347 | 0.525 | 1.411 | 4.302 |
| b_autop_th | -0.121 | -0.334 | 0.039 | 0.059 | 0.269 | 0.020 | 0.768 | 0.958 | 0.783 | 0.696 | 0.649 | 2.206 |
| b_autop_apt | -0.431 | -0.069 | -0.438 | -0.042 | -0.461 | -0.037 | 0.477 | 0.346 | 0.465 | 0.846 | 0.568 | 2.252 |
| b_p | 0.971 | 0.961 | 0.963 | 1.026 | 0.931 | 0.202 | 0.358 | 4.011 | 0.358 | 1.231 | 0.407 | 3.893 |
| b_numsale | 0.004 | 0.784 | 0.004 | 1.058 | 0.004 | 0.021 | 0.002 | 1.667 | 0.002 | 1.472 | 0.002 | 5.020 |
| mu_1 | 1.328 | 4.333 | 1.474 | 5.432 | 1.210 | 0.992 | 3.644 | 4.232 | 3.885 | 1.299 | 2.786 | 15.588 |
| mu_2 |  |  |  |  |  |  | 3.277 | 2.415 | 3.220 | 4.393 | 3.717 | 5.678 |
| LL(0) | -6077.24 | | -6077.24 | | -6077.24 | | -6077.24 | | -6077.24 | | -6077.24 | |
| LL(final) | -862.48 | | -862.27 | | -862.69 | | -858.51 | | -858.43 | | -858.15 | |
| Adj. $\rho^{2}$ | 0.8497 | | 0.8497 | | 0.8497 | | 0.8502 | | 0.8502 | | 0.8502 | |
